# Retraction Note: Dual roles of miR-374a by modulated c-Jun respectively targets CCND1-inducing PI3K/AKT signal and PTEN-suppressing Wnt/β-catenin signaling in non-small-cell lung cancer

**DOI:** 10.1038/s41419-021-03974-4

**Published:** 2021-07-07

**Authors:** Mengyang Zhao, Ping Xu, Zhen Liu, Yan Zhen, Yiyu Chen, Yiyi Liu, Qiaofen Fu, Xiaojie Deng, Zixi Liang, Yonghao Li, Xian Lin, Weiyi Fang

**Affiliations:** 1 Cancer Center, Integrated Hospital of Traditional, Chinese Medicine, Southern Medical University, Guangzhou, 510315 China; 2 Department of Oncology, The People’s Hospital of Zhengzhou University, Zhengzhou, Henan 450003 China; 3 Respiratory Department, Peking University Shenzhen Hospital, Shenzhen, 518034 China; 4 Key Laboratory of Protein Modification and Degradation, School of Basic Medical Sciences and Affiliated Cancer Hospital & Institute, Guangzhou Medical University, Guangzhou, 511436 China

Retraction to: *Cell Death & Disease*

10.1038/s41419-017-0103-7 published online 23 January 2018

The authors have retracted this article because there are anomalies in Figure 5: panel siJun/pc-9 in part F (migration assay) overlaps with panel pc-9/siJun in part G (invasion assay). All authors agree with this retraction.

